# The impact of financial incentives and restrictions on cyclical food expenditures among low-income households receiving nutrition assistance: a randomized controlled trial

**DOI:** 10.1186/s12966-021-01223-7

**Published:** 2021-12-04

**Authors:** Sruthi Valluri, Susan M. Mason, Hikaru Hanawa Peterson, Simone A. French, Lisa J. Harnack

**Affiliations:** 1 Division of Epidemiology & Community Health, School of Public Health, University of Minnesota, 1300 South 2nd Street, Suite 300, Minneapolis, MN 55454 USA; 2 University of Minnesota Medical School, Minneapolis, MN USA; 3 Department of Applied Economics, College of Food, Agricultural and Natural Resource Sciences, University of Minnesota, Minneapolis, MN USA

**Keywords:** Supplemental nutrition assistance program, Benefit cycle, Financial incentives, Financial restrictions, Cyclical food expenditures

## Abstract

**Background:**

The Supplemental Nutrition Assistance Program (SNAP) is the largest anti-hunger program in the United States. Two proposed interventions to encourage healthier food expenditures among SNAP participants have generated significant debate: financial incentives for fruits and vegetables, and restrictions on foods high in added sugar. To date, however, no study has assessed the impact of these interventions on the benefit cycle, a pattern of rapid depletion of SNAP benefits that has been linked to worsening nutrition and health outcomes over the benefit month.

**Methods:**

Low-income households not currently enrolled in SNAP (*n* = 249) received benefits every 4 weeks for 12 weeks on a study-specific benefit card. Households were randomized to one of four study arms: 1) incentive (30% incentive for fruits and vegetables purchased with study benefits), 2) restriction (not allowed to buy sugar-sweetened beverages, sweet baked goods, or candy using study benefits), 3) incentive plus restriction, or 4) control (no incentive or restriction). Weekly household food expenditures were evaluated using generalized estimating equations.

**Results:**

Compared to the control group, financial incentives increased fruit and vegetable purchases, but only in the first 2 weeks after benefit disbursement. Restrictions decreased expenditures on foods high in added sugar throughout the benefit month, but the magnitude of the impact decreased as the month progressed. Notably, restrictions mitigated cyclical expenditures.

**Conclusions:**

Policies to improve nutrition outcomes among SNAP participants should consider including targeted interventions in the second half of the month to address the benefit cycle and attendant nutrition outcomes.

**Trial registration:**

ClinicalTrial.gov, NCT02643576. Retrospectively registered December 22, 2014.

**Supplementary Information:**

The online version contains supplementary material available at 10.1186/s12966-021-01223-7.

## Background

The Supplemental Nutrition Assistance Program (SNAP) is the largest nutrition assistance program in the United States. In 2020, one in nine Americans—over 39 million adults and children—participated in SNAP every month. The program distributed over $74 billion in benefits over the course of the year [1]. Evidence suggests that SNAP participants have lower overall dietary quality, consume fewer fruits and vegetables, and purchase more calories from sugars compared to non-participants [2]. Adverse health outcomes associated with poor nutrition, such as type 2 diabetes and unhealthy weight, also impact SNAP participants at high rates [3]. In light of these data, there has been significant interest in interventions to promote healthy food purchases among SNAP participants.

Two commonly cited policy proposals to improve nutrition outcomes among SNAP participants are financial incentives to increase fruit and vegetable expenditures, and restricting the use of benefits on foods high in added sugar. A robust body of literature has shown that lowering the price of healthy foods shifts purchasing towards healthier options [4–6], reflecting the tendency of consumers to purchase more of a product as the price decreases (i.e., demand slopes curve downward). A meta-analysis of observational studies found that a 10% reduction in price was associated with a 12% increase in consumption of healthy foods [7]. A separate review found that a 10% reduction in price was associated with a 7 and 6% increase in spending for fruits and vegetables, respectively [8]. Based on these estimates of price elasticities of demand (i.e., the percentage change in purchases with a percentage change in price), a 30% reduction in prices for fruits and vegetables is estimated to increase spending across both food groups by 19% [9]. To that end, the United States Department of Agriculture (USDA) Healthy Initiatives Pilot study evaluated a 30-cent incentive for every SNAP dollar spent on targeted fruits and vegetables [9]. The study found that incentives increased intake of fruits and vegetables by 26% [10] and expenditures by 8.5% [11]. The positive impact of incentives on fruit and vegetable expenditures and intake has been supported by subsequent trials [12–16].

The effect of restrictions on foods high in added sugar has been less extensively studied. Restricting the purchase of foods using benefits effectively leads to a decrease in SNAP purchasing power [17, 18], though there is significant debate on which foods should be restricted. Some proponents argue for the ban of limited food categories such as sugar-sweetened beverages [19], while others advocate for restrictions on a broader range of unhealthy foods such as salty snack foods, fatty meat and dairy products, or foods prepared with partially hydrogenated oils [20]. Important ethical implications also arise from such policy proposals, including the potential for stigmatizing or penalizing low-income households [19]. Regardless of the types of foods restricted, households can offset restrictions on SNAP benefits by using their own income to purchase restricted items [21]. Thus, the extent to which restrictions lead to a corresponding decrease in purchases and consumption of restricted items remains unclear. Previous studies based on our research group’s data found that, on average, restrictions on foods high in added sugar were associated with reductions in intake [15] and expenditures on restricted foods [16].

However, while both proposed reforms have generated robust discussion [17], there has been no evaluation of how they may affect a phenomenon of significant public health concern known as the “benefit cycle,” a pattern of rapid depletion of benefits among households participating in SNAP. On average, SNAP households spend over half their benefits within a week of benefit distribution; a quarter of households exhaust benefits within the first week [22, 23]. Prior research has hypothesized that the benefit cycle is due to time inconsistent preferences, which suggests that households demonstrate a strong present-bias and preference for immediate consumption of SNAP benefits after distribution [24, 25]. This results in a cyclical pattern in food expenditures, which may be associated with adverse nutritional and health outcomes. Evidence suggests that the benefit cycle aligns with deteriorating caloric intake [24, 26], worsening food insecurity [27], and reductions in fruit and vegetable intake and dietary quality over the course of the benefit month [26, 28]. SNAP participants may also be at greater risk for emergency room visits at the end of the month [29], including for hypoglycemic episodes [30], and hypertension-related emergencies [31].

Cyclical food expenditure patterns that characterize the benefit cycle present unique policy and research challenges relevant to the proposed interventions. In particular, the impact of proposed reforms may vary by timing in the month, which may not be captured by average effects that obfuscate continued problems with nutrition towards the end of the benefit month [32]. For example, financial incentives may increase fruit and vegetable expenditures immediately after benefit disbursement, but fail to address worsening dietary quality towards the end of the month. Likewise, the impact of restrictions on discouraging unhealthy purchases may also diminish over the course of the benefit month as households exhaust benefits that are subject to restriction and substitute other sources of income [25]. Ideally, both proposals would help smooth observed fluctuations in nutrition quality over the course of the month—either by expanding household budgets with incentives, or by restricting bulk purchasing of unhealthy food early in the month. To date, however, no study has assessed the impact of incentives, restrictions, or the combination of both proposals on the benefit cycle.

This paper aims to address this critical gap in the literature by using data from a randomized controlled trial of low-income households receiving nutrition assistance designed to mimic SNAP. The trial evaluated a 30% incentive on the purchase on fruits and vegetables, restrictions on the use of benefits for the purchase of foods high in added sugar, and a combination of both incentives and restrictions. We assessed the impact of these policies on expenditures on fruits and vegetables, foods high in added sugar, total food at home (FAH), and food away from home (FAFH) over the course of the benefit month.

## Methods

### Study design

The design and protocol details of this study have been described previously [15]. In brief, this was a 2 × 2 randomized factorial trial to evaluate the impact of financial incentives and restrictions among low-income households receiving nutrition assistance. The primary outcomes were dietary intake and nutritional quality. Low-income households currently not enrolled in SNAP were recruited between August 2013 and May 2015. After baseline measures were completed, households were enrolled in the study nutrition assistance program, which was modeled after SNAP. Participants received benefits every 4 weeks for 12 weeks through a study debit card. The amount of nutrition assistance was equal to the average SNAP monthly benefit amount in Hennepin and Ramsey Counties in Minnesota in June 2013 ($139, $233, $350, $421, $493 per month for households with 1, 2, 3, 4, and 5 or more members, respectively). Similar to SNAP, study benefits could not be used for alcoholic drinks, hot prepared foods, or in restaurants.

Households were randomized into one of four study arms: 1) incentive (30 cents for every benefit dollar spent on fruits and vegetables), 2) restriction (benefits could not be used for the purchase of sugar-sweetened beverages, sweet baked goods, or candies); 3) incentive-plus-restriction, and 4) control (no incentives or restrictions). For comparability, the choice of foods for incentives was based on the USDA HIP, which showed that a 30% incentive was both effective and feasible for large-scale expansion [9]. Restrictions targeted foods that were leading sources of added sugars and solid fats in the American diet and contributed minimally to vitamin and mineral intake. Together, the three identified food categories—sugar-sweetened beverages, sweet baked goods, and candies—account for more than half the added sugars in the average American diet, and one-tenth of the calories and solid fats consumed by Americans [33, 34]. Additional file 1 provides descriptions of each of the experimental arms and targeted foods in greater detail. The trial protocol is described in Additional file 2.

### Study population

Low-income households in the Minneapolis-St. Paul, Minnesota, metropolitan area were recruited using study fliers posted in community locations in neighborhoods with high poverty rates; distributing fliers through food pantries; and referrals from organizations that serve low-income households. Eligibility criteria were established to recruit adults who were near eligible or eligible for SNAP but not currently enrolled. Criteria included: 1) not currently enrolled in SNAP or planning to enroll during the study; 2) household income at or below 200% the federal poverty level or participating in a government program that qualifies households for SNAP; and 3) adult primarily responsible for food shopping is able to read and speak English. Some SNAP eligibility criteria, such as asset tests, were not applied. The study sample was calculated to detect a linear trend of 4.5% declining change in energy intake per study arm, with 80% power, 2-tailed 5% *α* error, and a pre-post test correlation of 0.8. The analytic sample was initially assumed to be 300, but revised to 280 households due to a reduction in study budget. The University of Minnesota Institutional Review Board approved all aspects of the study (ClinicalTrials.gov: NCT02643576). The Consolidated Standards of Reporting Trials (CONSORT) and the template for intervention description and replication (TIDieR) checklists were used for reporting this study (see Additional files 3 and 4, respectively).

### Measures

At the baseline visit, participants completed a survey that included questions about demographic characteristics of the primary respondent, and household composition. Household resources were evaluated using annual household income; car ownership; employment status; health status of the primary respondent; participation in the Special Supplemental Nutrition Program for Women, Infants, and Children (WIC) at the time of study enrollment; and use of community food assistance (CFAs) programs (food banks, emergency soup kitchens, or delivery programs) in the month prior to study enrollment. Household food security in the month prior to study enrollment was evaluated using the USDA six-item short form food security module, which categorizes households as having high/marginal, low, or very low food security [35].

Household expenditures were evaluated using a simple annotated receipt method [36]. Participants were asked to collect and submit all household receipts for food and beverage purchases on a weekly basis. Items with unclear descriptions required annotation (e.g., “produce” is annotated to specify “bananas”). Research staff ensured completeness of receipt submission using the online system associated with the study debit card, which monitored and tracked expenditures made with benefits. Staff were therefore able to cross-reference submitted receipts with the online log of expenditures, contact participants for expenditures missing receipts, and verify weeks without any expenditures.

Expenditures were categorized as food at home (FAH) or food away from home (FAFH). FAH expenditures include purchases at retailers that primarily sell unprepared foods (e.g., grocery stores, drug stores, warehouse club stores). FAH expenditures were further disaggregated into two food categories: fruits and vegetables, and foods high in added sugar (sugar-sweetened beverages, sweet baked goods, and candy). FAFH expenditures include purchases made at vendors that primarily sell prepared foods (e.g., full- or limited-service restaurants). Timing of food expenditures within the benefit month was determined using receipt dates correlated with benefit disbursement. Day 1 refers to the day of benefit distribution, and day 28 refers to the last day of the benefit month.

### Statistical analysis

Analyses for this paper included 2 months of food expenditure data from the intervention period in order to capture typical food purchasing behavior in the presence of nutrition assistance. The final month of the study was excluded from analyses due to evidence of participants cashing out benefits earlier in the final month of the study, which was likely triggered by the study coming to an end. Because loss to follow-up was low in this sample, only households that submitted receipts every week were included to ensure completeness of data across the benefit months.

Linear regression models were constructed using generalized estimating equations (GEEs) and Huber-White robust standard errors with unstructured correlation matrices. The outcomes of interest were expenditures in US dollars. Separate models were specified for expenditures on fruits and vegetables, foods high in added sugar, and total FAH. We also specified a model for FAFH to evaluate any changes in food expenditures that were not direct targets of the policy interventions. Expenditures were aggregated by week since households do not shop for food every day, and daily data consisted of many days with zero expenditures. For each month, weeks 1, 2, 3 and 4, were comprised of days 1 through 7, 8 through 14, and 15 through 21, and 22 through 28, respectively. Expenditures made beyond day 28 were considered part of week 4 if new benefits had not been distributed, or week 1 of the subsequent month if the next month’s benefits had been distributed. Thus, timing in the benefit month was evaluated by week, with values 1 to 4. A two-way interaction term between the variables for week and study arm was included to assess whether food purchasing behavior varied by week, and whether this pattern differed by the household’s assigned experimental arm.

Based on previous studies showing that resource constraints may impact cyclical food expenditures, models controlled for the following household resources: annual household income (less than $14,999, $15–$24,999, $25–$34,999, and more than $35,000), car ownership, unemployment, concurrent WIC participation, baseline household food security status, use of any CFA programs, and health status (diagnosis of hypertension, diabetes, cholesterol, or other heart disease) to account for healthcare costs. Models were also adjusted for household composition (household headed by single adult, number of children in the household) and demographic characteristics of the primary shopper (age, gender, and college education or more). Race and ethnicity of the primary respondent were included as proxy variables for historical and current systemic racism [37] that shapes a household’s access to economic resources, ability to accrue financial resources [38], and availability of healthy food environments [39, 40]. Finally, all models controlled for baseline weekly expenditures, and the calendar month associated with expenditures to account for any seasonal trends.

Adjusted mean expenditures and 95% confidence intervals for each intervention arm were calculated using parameter estimates from the final regression models. To better visualize patterns in food purchasing, predictive margins were plotted for fruits and vegetables, foods high in added sugar, and total FAH. Analyses was conducted between 2019 and 2020 in Stata, version 16.1 (Stata-Corp LP, College Station, TX).

#### Study results

**Fig. 1:**
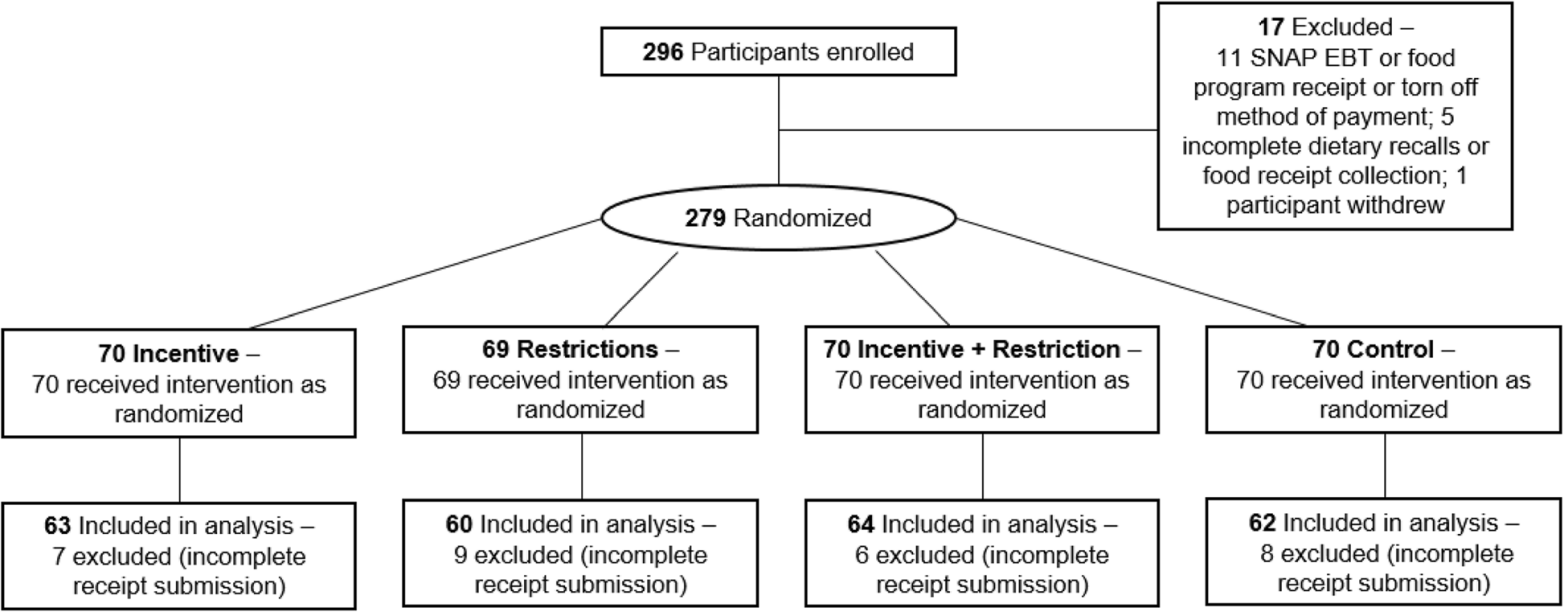
CONSORT flow diagram. *Note*: SNAP = Supplemental Nutrition Assistance Program; EBT = Electronic Benefits Transfer

Of the 279 participants randomized at baseline, 249 participants submitted receipts every week and were included in analyses (Fig. 1). There were no significant differences in the demographic characteristics of households with and without complete weekly data (data not shown). Table 1 presents household characteristics of the final sample. Most participants were women (81%), with an average age of 44.7 years. A third of households reported annual household incomes below $14,999, 54% were unemployed, 30% did not own a car, and 46% reported very low food security. There were no differences in household characteristic by study arm (Table 1). Participants submitted a total of 13,944 household-days of expenditure data. Additional file 5 presents unadjusted average daily expenditures of the study sample.

**Table 1:** Baseline characteristics of households receiving monthly nutrition assistance, by study group (*n* = 249)

		n (%)				
Characteristic		Incentive (*n* = 64)	Restriction (*n* = 60)	Incentive + Restriction (*n* = 63)	Control (*n* = 62)	*p*
Age, years ($$\overline{x}$$, SD)		42.5 (1.54)	44.7 (1.87)	47.37 (1.55)	44.3 (1.65)	0.21
Female		53 (82.8)	49 (81.7)	50 (79.4)	50 (80.7)	0.97
Non-Hispanic Black		33 (51.6)	35 (58.3)	27 (42.9)	32 (51.6)	0.39
Hispanic		13 (20.3)	25 (18.3)	10(15.9)	10 (16.1)	0.91
Some college or more		48 (75.0)	37 (61.7)	47 (74.6)	45 (72.6)	0.34
Single adult		28 (43.8)	29 (48.3)	29 (46.0)	26 (41.9)	0.90
Number of children ($$\overline{x}$$, SD)		1.3 (0.15)	1.3 (0.18)	1.0 (0.16)	1.1 (0.18)	0.64
Annual household income						
	$14,999 or less	20 (33.9)	22 (42.3)	15 (25.4)	20 (35.1)	0.52
	$15,000 - $24,999	16 (27.1)	10 (19.2)	14 (23.7)	12 (21.1)	
	$25,000 - $34,999	12 (20.3)	15 (28.9)	15 (25.4)	13 (22.8)	
	$35,000 or more	11 (18.6)	5 (9.6)	15 (25.4)	12 (21.1)	
Unemployed		30 (46.9)	34 (56.7)	33 (52.4)	37 (59.7)	0.51
Car owner		47 (73.4)	42 (70.0)	42 (66.7)	43 (69.4)	0.88
Adverse health status		24 (37.5)	20 (33.3)	31 (49.2)	25 (40.3)	0.33
Household food security						
	Very low	29 (45.3)	28 (46.7)	30 (47.6)	27 (43.6)	0.24
	Low	28 (43.8)	20 (33.3)	20 (31.8)	17 (27.4)	
	High or marginal	7 (10.9)	12 (20.0)	13 (20.6)	18 (29.0)	
WIC participant		8 (12.5)	6 (10.0)	5 (7.9)	11 (17.7)	0.39
CFA program client		25 (39.1)	22 (36.7)	22 (34.9)	25 (40.3)	0.93

*Note*: Adverse health status is defined as hypertension, diabetes, cholesterol, or other heart disease. WIC is the Special Supplemental Nutrition Program for Women, Infants, and Children; CFA is community food assistance (includes food banks, emergency soup kitchens, and meal delivery services)

### Fruits and vegetables

**Fig. 2:**
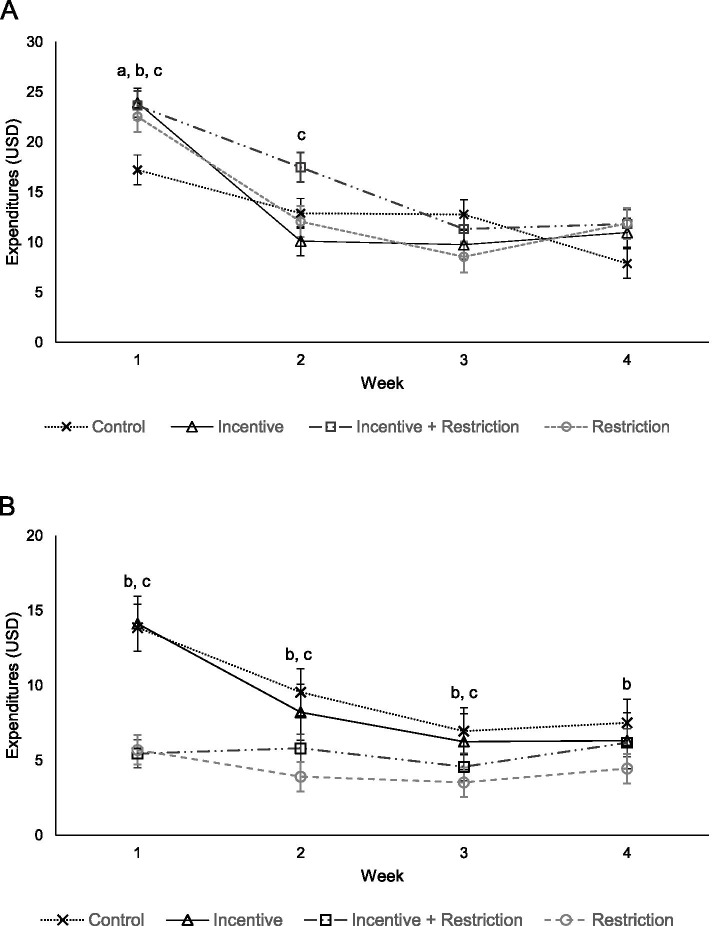
Adjusted mean weekly household expenditures by study group for **A**) fruits and vegetables and **B**) foods high in added sugar (*n* = 1992 household-weeks). *Note*: Statistically significant differences between control group and ^a^Incentive group, ^b^Restriction group, and ^c^Incentive + Restriction group (*p* < 0.05)

Fruit and vegetable expenditures varied by study arm in the first 2 weeks of the of the benefit month (Fig. 2A). Compared to the control group, the incentive arm spent $6.72 (95% Confidence Interval [CI]: $2.64, $10.80) more on fruits and vegetables in the first week of the benefit month (Additional file 6). The restriction group spent $5.32 (95% CI: $1.12, $9.53) more than the control group in the first week. Finally, the incentive-plus-restriction group spent $6.44 (95% CI: $2.36, $10.51) and $4.60 (95% CI: $1.53, $8.67) more than the control group in the first and second weeks, respectively. Fruit and vegetable expenditures were cyclical for all study groups, with a decline in expenditures in the second week that was maintained for the remainder of the benefit month (*p* < 0.001, respectively; see additional file 7 for joint test for expenditure smoothing).

### Foods high in added sugar

Expenditures on foods high in added sugar differed for intervention groups throughout the benefit month (Fig. 2B). Compared to the control group, the restriction group spent $8.17 (95% CI: -$10.84, −$5.50), $5.63 (95% CI: -$8.31, −$2.96), $3.42 (95% CI: -$6.11, −$1.74), and $3.05 (95% CI: -$5.73, −$0.74) less compared to the control group in weeks 1 through 4, respectively (Additional file 6). The incentive-plus-restriction group spent $8.42 (95% CI: -$11.01, −$5.83) and $3.73 (95% CI: -$6.31, −$1.41) less on foods high in added sugar compared to the control group in weeks 1 and 2, respectively. Notably, the restriction and incentive-plus-restriction groups did not demonstrate cyclical food expenditures, while expenditures were cyclical for the incentive and control groups (*p* < 0.001, respectively; Additional file 7).

### Total FAH and FAFH

**Fig. 3:**
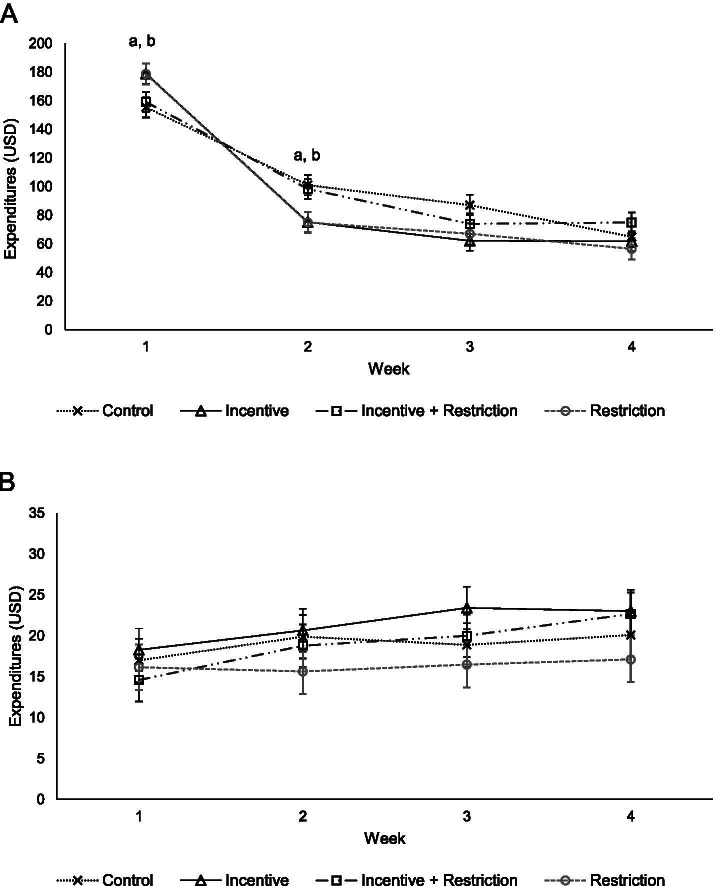
Adjusted mean weekly household expenditures by study group for **A**) total food at home (FAH) and **B**) food away from home (FAFH) (*n* = 1992 household-weeks). *Note:* Statistically significant differences between control group and ^a^Incentive group, ^b^Restriction group, and ^c^Incentive + Restriction group (*p* < 0.05)

Both the incentive and restriction groups demonstrated a more severe cyclical pattern in total FAH spending compared to the control group (Fig. 3A). The incentive group spent $23.50 (95% CI: $3.86, $43.15) more in the first week compared to the control group, but spent $25.82 (95% CI: -$45.47, −$6.18) and $24.96 (95% CI: − 44.61, −$5.32) less in weeks 2 and 3, respectively (Additional file 6). The restriction group spent $23.20 (95% CI: $2.92, $43.47) more on total FAH in the first week compared to the control group. In the second and third weeks, however, the restriction group spent $26.17 (95% CI: -$46.44, −$5.90) and $20.03 (95% CI: -$40.30, $2.39) less compared to the control group, respectively. There were no significant group differences in expenditures in the final week of the benefit month. Nevertheless, total FAH expenditures were cyclical for all study groups (Additional file 6). Finally, analysis of FAFH expenditures showed that FAFH expenditures did not vary over the benefit month by study group (Fig. 3B); there was also no evidence of cyclical FAFH spending (Additional file 7).

## Discussion

Financial incentives and restrictions to encourage healthier food expenditures among SNAP households have generated significant discussion. To our knowledge, the present study is the first to evaluate the impact of both types of interventions on cyclical food expenditures over the course of the benefit month. We found significant differences in the impact of proposed program reforms depending on the week of the month. For fruits and vegetables, financial incentives had the intended effect of increasing expenditures. Households in the restriction group also increased fruit and vegetable spending, which may be due to a shift of resources that would otherwise be spent on restricted foods. However, the impact of financial incentives was limited to the first 2 weeks after benefit disbursement. Notably, financial incentives did not help mitigate the reduction in fruit and vegetable expenditures towards the end of the month, an aspect of the benefit cycle that has been of great public health and policy concern [17]. Instead, fruit and vegetable expenditures were robustly cyclical for all study groups.

These findings add important nuance to results of previous research. The USDA Healthy Incentives Pilot study found that financial incentives increased both expenditures and consumption of targeted fruits and vegetables [10, 11]. Prior work based on our study data also demonstrated that, on average, financial incentives improved both expenditures and intake of fruits [15, 16]. However, we find that there may be significant intra-monthly variation in fruit and vegetable expenditures in response to financial incentives. While more frequent distribution of benefits has been proposed to help smooth household expenditures and improve the dietary quality of SNAP households, a recent study of low-income households in San Francisco found that weekly benefit distribution of food vouchers did not improve fruit and vegetable intake or dietary quality [41]. More targeted interventions in the second half of the benefit month may therefore be necessary to directly address the benefit cycle and the attendant cycles in nutrition and dietary quality. For example, an immediate 50% discount on fruit and vegetables at checkout was found to be effective in increasing fruit and vegetable expenditures of low-income households—though there was no discernable impact on intake [12]. The magnitude of the incentive and the diversity of foods selected for restriction may also impact the effectiveness of proposed policies. Our study incentive structure closely mimicked the USDA HIP for comparability, while the restricted items were selected based on the nutrient composition of the average American diet. An alternative combination of incentives and restrictions (e.g., 50% incentive and a greater number of restricted food groups) may have attenuated cyclical food purchasing behavior to a greater degree. A better understanding of the behavioral (e.g., how households use different sources of income) [25] or contextual (e.g., food prices, quantities, transportation, perishability) factors [42, 43] underlying cyclical patterns in fruit and vegetable expenditures is necessary to develop the most cost-effective combination of policy interventions to mitigate cyclical fruit and vegetable expenditures and worsening dietary quality. Importantly, additional research is necessary to gain insights from SNAP participants on approaches that may help them smooth fruit and vegetable expenditures and to test such approaches [44].

For foods high in added sugar, restrictions both decreased expenditures and mitigated the cyclical patterns in expenditures. The restriction group spent less on foods high in added sugar than the control group throughout the benefit month, though it is worth noting that the magnitude of the difference between groups declined substantially over the course of the month. Similarly, the incentive-plus-restriction group spent less than the control group for the first 2 weeks. Notably, both the incentive and incentive-plus-restriction groups smoothed purchasing of foods high in added sugar between disbursement dates. In contrast, study groups without restrictions (i.e., the incentive and control groups) demonstrated cyclical expenditure patterns for foods high in added sugar. Prior evaluations based on our study data found that, on average, restrictions reduced both consumption and intake of sugar-sweetened beverages and sweet baked goods [15, 16]. Our findings suggest that restrictions—either alone or in tandem with incentives—may also mitigate cyclical food purchasing for foods high in added sugar. Additional research is necessary to evaluate whether these changes in expenditures indicate a change in cyclical intake over the course of the benefit month.

Total FAH expenditures were cyclical for all study groups, but there were group differences by week. The incentive and restriction groups demonstrated a more severe fluctuation in overall spending compared to the control group, spending more in the first week but less in the subsequent 2 weeks. These differences may be explained by the patterns observed for fruits and vegetables. However, since we did not disaggregate expenditures for all food types, there may be unobserved changes in other food categories. Additional research is necessary to clarify the full impact of proposed program modifications beyond the intended targets of the proposals. Taken together, findings from this study suggest that evaluations of SNAP households—or more broadly, low-income households receiving nutrition assistance in a monthly lump sum—should consider the timing of outcome evaluation. For example, a survey of food expenditures or intake immediately after benefit disbursement may overestimate overall effects, while surveys at the end of the benefit month may underestimate effects.

### Limitations

This study has several limitations. Although the study intervention closely mimics SNAP, there are several key differences. The study eligibility criteria were less restrictive than state-wide standards for Minnesota SNAP. This resulted in a sample of households with more resources than SNAP participants—the average household income in our sample was $26,000 compared to $21,100 among Minnesota SNAP enrollees in 2015 [45]. Nonetheless, this limitation likely yielded more conservative estimates since evidence suggests more resource constrained households experience more severe cycles in food expenditures [46, 47]. Furthermore, the study sample was recruited from the Minneapolis-St. Paul, Minnesota, metropolitan area. Among the study participants, 51 and 18% of households identified as Black and Hispanic, respectively, compared to 25 and 4% of Minnesota SNAP enrollees [45] and 28 and 24% in a national sample of SNAP participants [22]. Our study sample was also more educated, with 71% of participants indicating they had some college education or more, compared to 15.6 and 38% among a sample of Minnesota and national SNAP enrollees, respectively [22, 45]. Benefit amounts were also based on SNAP averages for household size and not the benefit algorithm employed by the USDA. Thus, findings of this study may not generalize to the SNAP participants. It is worth noting, however, that the magnitude and pattern of the benefit cycle in our study sample is very similar to data reported by the USDA among SNAP households [22, 23]. Evidence also suggests that cyclical food expenditures among SNAP participants is not associated with demographic characteristics such race, ethnicity, or education [25]. Instead, cyclical food expenditures appear sensitive to the timing of income receipt regardless of the source of income—whether it is SNAP benefits [22], Social Security [48, 49], pay checks [50–52], or study benefits as demonstrated in this study.

Household food purchasing behavior may also be influenced by the novelty of the intervention itself. However, we analyzed 2 months of household food expenditures and found that expenditures were robustly cyclical throughout. This aligns with prior research that suggests SNAP households have cyclical expenditure patterns throughout their enrollment in the program [22]. The study also relies on households submitting food receipts. While research staff were able to track benefit expenditures in real-time, expenditures made using other income sources could not be verified. This may have resulted in underestimated expenditures if receipt submission was incomplete, which is of particular importance for foods high in added sugar since households may have used other income sources for restricted items. The study was also not powered to detect significance in secondary outcomes. Additional research is necessary to clarify how households use different income sources in response to incentives and restrictions. Finally, these findings are restricted to expenditures. While evidence suggests that expenditures align with intake over the course of the benefit month [26–28, 53], research is necessary to verify the impact of policy proposals on intra-monthly variations in nutrition outcomes.

## Conclusions

This study speaks directly to current research and policy discussions on interventions to encourage healthier food expenditures among SNAP participants. Our findings suggest that intra-monthly variations should be considered when crafting and evaluating SNAP policy reforms. We find that the impact of financial incentives to encourage healthier food purchasing behavior is limited to the first half of the benefit cycle. In contrast, restrictions reduced expenditures on food high in added sugar throughout the month, though the magnitude of the impact of restrictions declined over the course of the month. Notably, restrictions mitigated cyclical patterns in food expenditures for foods high in added sugar. Targeted interventions specific to the second half of the month may be necessary to address the benefit cycle and the resultant nutrition outcomes.

## Supplementary Information

**Additional file 1.** Description of experimental conditions

**Additional file 2.** Trial protocol.

**Additional file 3.** CONSORT checklist

**Additional file 4.** TIDieR checklist

**Additional file 5 **Unadjusted average daily household expenditures on food at home (FAH) and food away from home (FAFH) (*n* = 13,944 household-days)

**Additional file 6.** Estimated relationship between experimental condition, week in benefit month, and food expenditures

**Additional file 7 **Adjusted mean food expenditures by study group and week (*n* = 1992 household-weeks)

## Data Availability

The datasets used and/or analyzed during the current study are available from the corresponding author on reasonable request.

## Abbreviations

SNAP: Supplemental Nutrition Assistance Program
USDA: United States Department of Agriculture
WIC: Special Supplemental Nutrition Program for Women, Infants, and Children
CFA: Community food assistance
