# Supplementary material for: The impact of financial incentives and restrictions on cyclical food expenditures among low-income households receiving nutrition assistance: a randomized controlled trial
Source: Int J Behav Nutr Phys Act. 2021 Dec 4;18:157. doi: 10.1186/s12966-021-01223-7 (PMC8642917; doi:10.1186/s12966-021-01223-7)
Supplement: Supplementary file 1 — Additional file 1. Description of experimental conditions [file 12966_2021_1223_MOESM1_ESM.docx]

**Additional file 1.** Description of experimental conditions

|  | Experimental condition | | | |
| --- | --- | --- | --- | --- |
|  | Incentive | Restriction | Incentive + Restriction | Control |
| *Ineligible purchases*: alcoholic beverages, restaurant foods, and dietary supplements with debit card (similar to SNAP) | Yes | Yes | Yes | Yes |
| *Restrictions*: Sugar-sweetened beverages (water-based beverages with added sugar such as soft drinks, fruit drinks, energy drinks, and sports drinks), candy of all types, and prepared sweet baked goods (e.g., pies, cakes, cookies) | No | Yes | Yes | No |
| *30% incentive on eligible fruits and vegetables*:^a^ fresh fruits; frozen or dried fruits without sugar: fresh vegetables excluding white potatoes; frozen, canned or dried vegetables with no sauces or fat; and dry and canned mature beans | Yes | No | Yes | No |

^a^ Ineligible fruits and vegetables include fruits juices; canned, frozen, or dried fruits with sugar/syrup; canned or frozen vegetables with a sauce; pickled vegetables; and white potatoes.
