# Supplementary material for: The impact of financial incentives and restrictions on cyclical food expenditures among low-income households receiving nutrition assistance: a randomized controlled trial
Source: Int J Behav Nutr Phys Act. 2021 Dec 4;18:157. doi: 10.1186/s12966-021-01223-7 (PMC8642917; doi:10.1186/s12966-021-01223-7)
Supplement: Supplementary file 2 — Additional file 2. Trial protocol. [file 12966_2021_1223_MOESM2_ESM.doc]

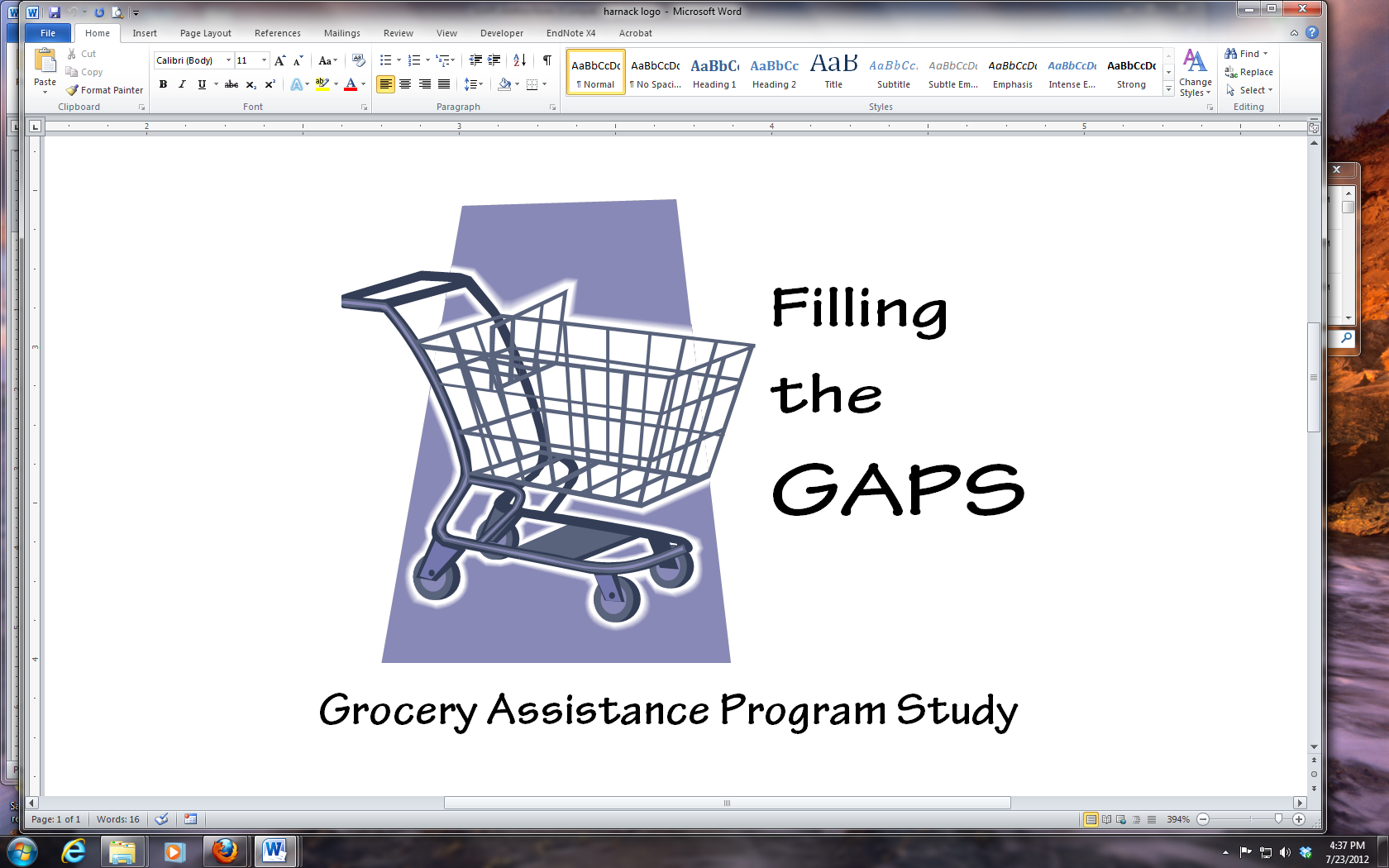


**Overall Study Protocol**

***Recruitment:***

Individuals who are responsible for the majority of their household’s food shopping who meet eligibility criteria will be targeted for recruitment via fliers posted at community locations in Minneapolis, via Craig’s list adverts, and through community and/or church newsletters. Prospective participants will contact the Project Coordinator via phone or email if they are interested. Up to 320 individuals will be recruited in order to obtain our desired number of study participants (n=300).

***Determining Eligibility:***

Upon contact with the Project Coordinator, the main food purchaser of the household will be read the Telephone Recruitment Screener Script. If the person is interested and eligible, a study packet will be mailed to him/her. This packet will contain a cover letter, a study summary, and a copy of the consent forms for his/her review. People will be given the opportunity to sign up for the study at the time of the screening phone call or receive a call back three days later.

If the person chooses a call-back, a follow up phone call will be made three days later to ensure delivery of study materials, to answer any questions, and to find out whether or not the participant has decided to participate. If the person is not interested, this should be recorded on the Telephone Recruitment Screener and s/he be thanked for their interest.

If the person is interested in participating, a baseline data collection appointment will be scheduled. The person will be sent a survey-visit confirmation letter and directions to the survey site (either a nearby community location or the ECRC). The person will also be told that the consent form mailed to them can be completed prior to or at the time of the survey visit.

***Baseline Data Collection, and Incentive Structure:***

**Survey Visit:**

Consent forms will be signed by the participant at the baseline data collection visit, along with the completion of the study questionnaire and anthropometry measurements. Participants will also be given instructions on how to collect food receipts for the following four weeks (and necessary materials) and be given a brief orientation to the 24-hour recall process. Each participant will be given a $15 Target Gift Card for the baseline data collection visit.

Note: this visit will be conducted within a four week window prior to the start date of the 16 week receipt collection portion of the study.

**Three, 24-hr recalls:**

Participants will then be asked to complete three, unannounced 24-hour dietary recalls over the telephone within the next two weeks. Upon completion of this task, they will be mailed a $40 Target Card.

*(Note: if 1/3 of the recalls are completed, the participant will receive a $10 card; if 2/3 recalls are complete, a $20 card will be provided; 3/3 recalls results in a $40 card).*

***Baseline Receipt Collection (Study Weeks 1-4)*:**

Starting at a set date, all participants within a particular wave will commence Week 1 of the ‘16 week receipt collection portion’ of the study. An email or text will be sent to all participants of a particular wave reminding them of this and to start collecting receipts.

Upon completion of the four weeks of receipt collection, a $30 Target Card will be mailed to the study participant.

***Randomization:***

All participants will be randomized to one of four, ‘Grocery Assistance Program’ treatment groups. The program will last for 12 weeks.

Participants will be mailed a set of instructions regarding their experimental condition (i.e. what types of groceries they may purchase with their GAPS Visa card). A follow-up phone call or meeting (depending upon participant preference) will be set up to explain the Grocery Assistance Program to which they were randomized.

In an effort to avoid unnecessary confusion, only the experimental condition to which the person is assigned will be explained. If a participant would like to know more, see the FAQs at the end of this document.

The four experimental conditions are as follows (See Experimental Procedures document for more information):

1. GAP Standard: Control condition- restrictions like those in SNAP
2. GAP Plus: Restrictions like those in SNAP plus sugar sweetened beverages, sweet baked goods, and candy restricted.
3. GAP Rewards: Restrictions like those in SNAP and 30% bonus for fruit and vegetable purchases (a bonus of 30% of their total monies spent on fruit and vegetables).
4. GAP Plus Rewards: Restrictions like those in SNAP plus sugar sweetened beverages, sweet baked goods, and candy restricted and 30% bonus for fruit and vegetable purchases.

***One day prior to commencing the ‘Grocery Assistance Program’:***

An email or text will be sent to the participant letting them know grocery funds will be available on their Visa card the following day.

*Note: If USPS is the preferred method of contact, a letter will be mailed four days in advance with the date funds will be available.*

***Grocery Assistance Program: Weeks 5-8***

Participants will collect all required food/beverage receipts and mail them back weekly. Upon receipt of the first four weeks of receipts, a $30 Target card will be mailed to the participant.

*(Note: if 1/4 of the weeks are collected, participants will receive a $5 card; if 2/4 are collected, a $10 card will be provided; if 3/4 are collected, a $15 card will be provided; 4/4 weeks results in a $30 card).*

Those participants randomized to the fruit and vegetable discount group will have their F&V discount totals calculated and added to their account on a consistent basis, provided the receipts to make this calculation are returned in a timely manner.

Any remaining money on the Visa card at the end of week four will be carried forward to the next four weeks.

***Grocery Assistance Program: Weeks 9-12***

Participants will collect all required food/beverage receipts and mail them back weekly. Upon receipt of the next four weeks of receipts, a $30 Target card will be mailed to the participant.

*(Note: if 1/4 of the weeks are collected, participants will receive a $5 card; if 2/4 are collected, a $10 card will be provided; if 3/4 are collected, a $15 card will be provided; 4/4 weeks results in a $30 card).*

Those participants randomized to the fruit and vegetable discount group will have their F&V discount totals calculated and added to their account on a consistent basis, provided the receipts to make this calculation are returned in a timely manner.

Any remaining money on the Visa card at the end of week eight will be carried forward to the final four weeks.

***Grocery Assistance Program: Weeks 13-16***

Participants will collect all required food/beverage receipts and mail them back weekly. Upon receipt of the last four weeks of receipts, a $30 Target card will be mailed to the participant (or given to them at the follow-up data collection visit).

*(Note: if 1/4 of the weeks are collected, participants will receive a $5 card; if 2/4 are collected, a $10 card will be provided; if 3/4 are collected, a $15 card will be provided; 4/4 weeks results in a $30 card).*

Those participants randomized to the fruit and vegetable discount group will have their F&V discount totals calculated and added to their account on a consistent basis, provided the receipts to make this calculation are returned in a timely manner.

At the end of week 12, all Visa cards will be deactivated. Any remaining money on the cards will be returned to the study. Those participants due a bonus at the end of week 12 will be awarded these extra monies in the form of a Target Card.

***Final Data Collection, and Incentive Structure:***

**Survey Visit:**

Participants will complete the follow-up study questionnaire and have their weight measured. Participants will also be given an additional food amounts booklet, if needed, in order to conduct their final three 24-hour recalls. Each participant will be given a $15 Target Gift Card for the final data collection visit.

**Three, 24-hr recalls:**

Participants will be asked to complete three, unannounced dietary recalls over the next two weeks. Upon completion of this task, they will be mailed a $40 Target Card.

*(Note: if 1/3 of the recalls are completed, the participant will receive a $10 card; if 2/3 recalls are complete, a $20 card will be provided; 3/3 recalls results in a $40 card).*

***Frequently Asked Questions:***

If a participant requests to know more about the four different experimental conditions, they will be told:

*We are examining four different types of grocery assistance programs, each varying in the types of foods that are included as allowable purchases.*
