# Supplementary material for: The impact of financial incentives and restrictions on cyclical food expenditures among low-income households receiving nutrition assistance: a randomized controlled trial
Source: Int J Behav Nutr Phys Act. 2021 Dec 4;18:157. doi: 10.1186/s12966-021-01223-7 (PMC8642917; doi:10.1186/s12966-021-01223-7)
Supplement: Supplementary file 5 — Additional file 5 Unadjusted average daily household expenditures on food at home (FAH) and food away from home (FAFH) (n = 13,944 household-days) [file 12966_2021_1223_MOESM5_ESM.docx]

**Additional file 5.** Unadjusted average daily household expenditures on food at home (FAH) and food away from home (FAFH) (*n*=13,944 household-days)
