# Supplementary material for: The impact of financial incentives and restrictions on cyclical food expenditures among low-income households receiving nutrition assistance: a randomized controlled trial
Source: Int J Behav Nutr Phys Act. 2021 Dec 4;18:157. doi: 10.1186/s12966-021-01223-7 (PMC8642917; doi:10.1186/s12966-021-01223-7)
Supplement: Supplementary file 6 — Additional file 6. Estimated relationship between experimental condition, week in benefit month, and food expenditures [file 12966_2021_1223_MOESM6_ESM.docx]

**Additional file 6.** Estimated relationship between experimental condition, week in benefit month, and food expenditures

|  | | **Fruits and vegetables** | | **Foods high in added sugar** | | **Total FAH** | | **FAFH** | |
| --- | --- | --- | --- | --- | --- | --- | --- | --- | --- |
| Week | |  |  |  |  |  |  |  |  |
|  | Week 1 | ref |  | ref |  | ref |  | ref |  |
|  | Week 2 | -4.33  (2.13) | * | -4.32  (1.28) | ** | -54.38  (12.78) | *** | 2.90  (3.32) |  |
|  | Week 3 | -4.45  (2.06) | * | -6.91  (1.27) | *** | -68.32  (12.02) | *** | 1.89  (3.11) |  |
|  | Week 4 | -9.34  (2.01) | *** | -6.36  (1.20) | *** | -90.49  (11.40) | *** | 3.12  (3.23) |  |
| Week*Study group | |  |  |  |  |  |  |  |  |
|  | Week 1*Control | ref |  | ref |  | ref |  | ref |  |
|  | Week 1*Incentive | 6.72  (2.08) | ** | 0.26  (1.32) |  | 23.50  (10.02) | * | 1.29  (3.76) |  |
|  | Week 1*Restriction | 5.32  (2.14) | * | -8.17  (1.36) | *** | 23.20  (10.35) | * | -0.82  (3.86) |  |
|  | Week 1*Incentive+Restriction | 6.44  (2.08) | ** | -8.42  (1.32) | *** | 3.74  (10.02) |  | -2.40  (3.74) |  |
|  | Week 2*Control | ref |  | ref |  | ref |  | ref |  |
|  | Week 2*Incentive | -2.77  (2.08) |  | -1.34  (1.32) |  | -25.82  (10.02) | * | 0.77  (3.76) |  |
|  | Week 2*Restriction | -0.83  (2.14) |  | -5.63  (1.36) | *** | -26.17  (10.34) | * | -4.25  (3.87) |  |
|  | Week 2*Incentive+Restriction | 4.60  (2.08) | * | -3.73  (1.32) | ** | -2.60  (10.02) |  | -1.09  (3.75) |  |
|  | Week 3*Control | ref |  | ref |  | ref |  | ref |  |
|  | Week 3*Incentive | -3.01  (2.08) |  | -0.70  (1.32) |  | -24.96  (10.02) | * | 2.90  (3.76) |  |
|  | Week 3*Restriction | -4.23  (2.14) |  | -3.42  (1.37) | ** | -20.03  (10.34) | * | -2.99  (3.86) |  |
|  | Week 3*Incentive+Restriction | -1.45  (2.08) |  | -2.38  (1.32) |  | -13.11  (10.02) |  | 2.57  (3.75) |  |
|  | Week 4*Control | ref |  | ref |  | ref |  | ref |  |
|  | Week 4*Incentive | 3.08  (2.08) |  | -1.19  (1.33) |  | -3.08  (10.02) |  | 2.57  (3.76) |  |
|  | Week 4*Restriction | 3.99  (2.14) |  | -3.05  (1.36) | * | -8.45  (10.34) |  | -3.00  (3.86) |  |
|  | Week 4*Incentive+Restriction | 3.94  (2.08) |  | -1.33  (1.32) |  | 10.04  (10.02) |  | 2.90  (3.76) |  |
| Annual household income | |  |  |  |  |  |  |  |  |
|  | $14,999 or less | ref |  | ref |  | ref |  | ref |  |
|  | $15,000 - $24,999 | 0.43  (1.15) |  | 1.05  (0.79) |  | 5.14  (3.17) |  | -0.42  (2.78) |  |
|  | $25,000 - $34,999 | -0.39  (1.19) |  | 0.64  (0.81) |  | 3.78  (3.27) |  | 1.00  (2.86) |  |
|  | $35,000 or more | 4.40  (1.39) | ** | 1.61  (0.95) |  | 6.03  (3.83) |  | 11.99  (3.38) | *** |
| Unemployed | | 1.55  (0.88) | * | -1.36  (0.60) | * | -0.12  (2.41) |  | -3.49  (2.11) |  |
| Car owner | | 1.09  (0.98) |  | -2.05  (0.67) | ** | -2.20  (2.68) |  | -2.43  (2.35) |  |
| Adverse health status | | -0.49  (0.96) |  | 0.61  (0.66) |  | 9.68  (2.63) | *** | 2.09  (2.31) |  |
| Household food security | |  |  |  |  |  |  |  |  |
|  | Very low | ref |  | ref |  | ref |  | ref |  |
|  | Low | 2.13  (0.95) | ** | -0.45  (0.64) |  | 1.55  (2.62) |  | 3.18  (2.58) |  |
|  | High or marginal | 2.72  (1.07) | *** | -1.54  (0.73) | * | 6.69  (2.94) | * | 5.42  (2.28) | ** |
| WIC participant | | 2.10  (1.37) |  | -1.02  (0.94) |  | -2.06  (3.77) |  | -6.53  (3.31) |  |
| Food bank client | | -1.77  (0.89) | * | -1.37  (0.61) | * | -3.37  (2.47) |  | -2.64  (2.15) |  |
| Some college or more | | 1.15  (0.98) |  | 0.83  (0.86) |  | 0.53  (2.68) |  | -1.07  (2.35) |  |
| Single adult | | -0.25  (1.26) |  | -0.18  (0.41) | * | -1.43  (3.46) |  | -0.48  (3.03) |  |
| Number of children | | 1.58  (0.59) | ** | -0.18  (0.41) |  | 3.86  (1.63) | * | 1.11  (1.43) |  |
| Age | | 0.05  (0.04) |  | -0.04  (0.03) |  | -0.05  (0.11) |  | -0.17  (0.10) |  |
| Female | | -1.52  (1.11) |  | 0.48  (0.76) |  | 0.44  (3.06) |  | -2.72  (2.69) |  |
| Non-Hispanic Black | | -3.17  (1.00) | *** | -0.01  (0.68) |  | 4.54  (2.74) |  | -4.28  (2.39) |  |
| Hispanic | | -2.26  (1.31) | * | -0.69  (0.89) |  | 1.31  (3.59) |  | 1.13  (3.17) |  |
| Benefit amount | | 0.03  (0.008) | *** | 0.02  (0.005) | ** | 0.29  (0.02) | *** | 0.01  (0.02) |  |
| Expenditure month | | -0.16  (0.0) |  | -0.11  (0.07) |  | -0.42  (0.28) |  | -0.17  (0.24) |  |
| Baseline weekly spending | | 0.36  (0.03) | *** | 0.23  (0.03) | *** | 0.28  (0.02) | *** | 0.23  (0.02) | *** |
| Constant | | 4.11  (3.74) |  | 12.80  (2.53) | *** | 54.66  (11.83) | *** | 20.64  (8.68) | * |
| Number of observations | | 1816 |  | 1816 |  | 1816 |  | 1816 |  |

*Note*: Standard errors in parentheses. ****p*< 0.001; ***p*< 0.01; **p*< 0.05
