# Supplementary material for: The impact of financial incentives and restrictions on cyclical food expenditures among low-income households receiving nutrition assistance: a randomized controlled trial
Source: Int J Behav Nutr Phys Act. 2021 Dec 4;18:157. doi: 10.1186/s12966-021-01223-7 (PMC8642917; doi:10.1186/s12966-021-01223-7)
Supplement: Supplementary file 7 — Additional file 7 Adjusted mean food expenditures by study group and week (n = 1992 household-weeks) [file 12966_2021_1223_MOESM7_ESM.docx]

**Additional file 7**. Adjusted mean food expenditures by study group and week (*n*=1,992 household-weeks)

|  |  | **Week 1** | **Week 2** | **Week 3** | **Week 4** | ***p*** |
| --- | --- | --- | --- | --- | --- | --- |
| *Fruits and vegetables* | | |  |  |  |  |
| Control | | 17.21 (14.32, 20.12) | 12.89 (9.99, 15.78) | 12.77 (9.87, 15.68) | 7.88 (4.98, 10.78) | <0.001 |
| Incentive | | 23.94 (21.09, 26.79) | 10.11 (7.27, 12.96) | 9.75 (6.90, 12.60) | 10.96 (8.10, 13.81) | <0.001 |
| Restriction | | 22.54 (19.50, 25.58) | 12.06 (9.02, 15.10) | 8.54 (5.50, 11.59) | 11.87 (8.83, 14.91) | <0.001 |
| Incentive+Restriction | | 23.65 (20.79, 26.51) | 17.48 (14.63, 20.34) | 11.32 (8.46, 14.17) | 11.82 (8.96, 14.68) | <0.001 |
| *Foods high in added sugar* | | |  |  |  |  |
| Control | | 13.86 (12.02, 15.70) | 9.54 (7.70, 11.38) | 6.95 (5.10, 8.80) | 7.50 (5.66, 9.34) | <0.001 |
| Incentive | | 14.12 (12.30, 15.93) | 8.20 (6.93, 10.02) | 6.25 (4.43, 8.06) | 6.31 (4.50, 8.12) | <0.001 |
| Restriction | | 5.69 (3.76, 7.62) | 3.91 (1.97, 5.84) | 3.52 (1.59, 5.46) | 4.45 (2.51, 6.38) | 0.40 |
| Incentive+Restriction | | 5.44 (3.63, 7.26) | 5.81 (3.99, 7.62) | 4.57 (2.75, 3.38) | 6.17 (4.36, 7.99) | 0.59 |
| *Total food at home (FAH)* | | |  |  |  |  |
| Control | | 155.44 (141.44, 169.44) | 101.06 (87.07, 115.05) | 87.12 (73.13, 101.11) | 64.95 (50.95, 78.95) | <0.001 |
| Incentive | | 178.94 (165.18, 192.71) | 75.24 (61.48, 89.00) | 62.16 (48.41, 75.92) | 61.87 (48.11, 75.62) | <0.001 |
| Restriction | | 178.63 (163.98, 193.29) | 74.90 (60.24, 89.55) | 67.09 (52.43, 81.75) | 56.49 (41.83, 71.16) | <0.001 |
| Incentive+Restriction | | 159.19 (145.14, 172.95) | 98.46 (84.70, 112.22) | 74.01 (60.25, 87.78) | 74.99 (61.22, 88.76) | <0.001 |
| *Food away from home (FAFH)* | | |  |  |  |  |
| Control | | 16.99 (11.77, 22.20) | 19.89 (14.67, 25.11) | 18.87 (13.66, 24.09) | 20.10 (14.89, 25.32) | 0.77 |
| Incentive | | 18.27 (13.14, 23.41) | 20.66 (15.53, 25.79) | 23.41 (18.28, 28.54) | 23.00 (17.87, 28.13) | 0.39 |
| Restriction | | 16.16 (10.69, 21.64) | 15.64 (10.16, 21.12) | 16.48 (11.00, 21.96) | 17.11 (11.63, 22.59) | 0.97 |
| Incentive+Restriction | | 14.59 (9.45, 19.73) | 18.79 (13.65, 23.94) | 20.00 (14.86, 25.14) | 22.67 (17.53, 27.81) | 0.09 |

*Note*: 95% confidence intervals in parentheses. Adjusted for age, gender, race, ethnicity, education, household headed by single adult, number of children in household, baseline household food security status, annual household income, car ownership, employment status, concurrent participation in WIC, use of community food assistance program in month prior to study enrollment, benefit amount, expenditure month, study group, and baseline weekly spending.
